# A Method for Predicting Trajectories of Concealed Targets via a Hybrid Decomposition and State Prediction Framework

**DOI:** 10.3390/s25123639

**Published:** 2025-06-10

**Authors:** Zhengpeng Yang, Jiyan Yu, Miao Liu, Tongxing Peng, Huaiyan Wang

**Affiliations:** College of Mechanical Engineering, Nanjing University of Science and Technology, Nanjing 210094, China; yzp_njust@njust.edu.cn (Z.Y.); lm221939@njust.edu.cn (M.L.); 221101024477@njust.edu.cn (T.P.);

**Keywords:** millimeter-wave sensor, trajectory prediction, optimized algorithm, target telemetry

## Abstract

Accurate trajectory prediction of concealed targets in complex, interference-laden environments present a formidable challenge for millimeter-wave sensor tracking systems. To address this, we propose a state-of-the-art autonomous prediction framework that integrates an Improved Sequential Variational Mode Decomposition (ISVMD) algorithm with an Extreme Learning Machine (ELM), synergistically optimized by the novel Red-billed Blue Magpie Optimizer (RBMO). The ISVMD enhances signal reconstruction by transforming noisy target echo signals into robust feature sequences, effectively mitigating the impacts of environmental disturbances and intentional concealment. Subsequently, the RBMO-optimized ELM leverages these feature sequences to predict the future trajectories of concealed targets with high precision. The RBMO further refines critical parameters within the ISVMD-ELM pipeline, ensuring adaptability and computational efficiency across diverse scenarios. Experimental validation using real-world data demonstrates that the RBMO-ISVMD-ELM approach surpasses state-of-the-art algorithms in both accuracy and robustness when predicting the trajectories of concealed ground targets, achieving optimal performance metrics under demanding conditions.

## 1. Introduction

In recent years, the rapid advancement of sensor technology has markedly enhanced the performance of sensor algorithms, particularly those developed for the swift prediction and tracking of ground moving targets. These sensors have emerged as indispensable tools across a wide range of applications, including disaster rescue [1,2,3] and battlefield situational awareness [4,5]. Nevertheless, the traditional algorithms employed for tracking ground targets with these sensors, such as Detect-Before-Track (DBT) [6,7] and Track-Before-Detect (TBD) [8,9], depend extensively on trajectory prediction to optimize tracking efficacy. In environments characterized by varying degrees of interference, the sensors’ estimation errors for the motion states of moving targets, including parameters such as speed, relative distance, and relative angle, tend to increase, resulting in diminished accuracy of tracking data. Consequently, the development of algorithms capable of precisely predicting target positions remains a formidable challenge. Moreover, current research has largely overlooked the correction of prediction input data accuracy. Increasing attention is now being directed toward exploring how to supply target prediction algorithms with precise input data to enhance the detection of target position information.

Researchers have developed various advanced signal processing techniques, including wavelet transform [10], wavelet packet transform [11], and Empirical Mode Decomposition (EMD) [12]. Among these, Variational Mode Decomposition (VMD) has gained significant attention for its straightforward structure and superior performance [13]. Unlike EMD-based methods, VMD avoids error accumulation, and its integration with non-recursive variational modes and other algorithms demonstrates strong robustness and stability [14,15]. Liu H et al. enhanced VMD, achieving better processing performance compared to other models [16]. Ma J H et al. introduced an improved VMD algorithm that incorporates adaptive wavelet thresholding and noise reduction, with experiments showing reduced signal fluctuations post-decomposition [17]. However, the traditional VMD algorithm’s sensitivity to parameter selection poses challenges. To address this, researchers like Zhang X et al. employed the grasshopper optimization algorithm to adaptively determine VMD parameters [18], while Yan X proposed a parameter optimization approach using the Cooperation Search Algorithm (CSA) [19]. Other methods, such as particle swarm optimization [20,21] and ant colony algorithms [22], have also been applied to fine-tune VMD parameters. Despite these advancements, VMD alone struggles to achieve optimal results for signal decomposition when used for estimation and prediction tasks.

After obtaining the accurate target observation data set by using the above method, the data set is converted into a time series as the input of the subsequent prediction algorithm. To predict the target’s trajectory using the time-series data set, common methods include the Extreme Learning Machine (ELM) [23], Long Short-Term Memory (LSTM) [24,25], Gated Recurrent Unit (GRU) [26], and Bidirectional RNN (BiRNN) [27], among others. ELM is a learning algorithm for a single hidden layer feed-forward neural network, particularly well suited for predicting the trajectories of maneuvering targets. References [28,29,30] applied this method to predict the trajectories of ships or UAVs after developing a mathematical model of their motion. However, due to the structural characteristics of the traditional ELM algorithm, key parameters must still be set manually based on empirical experience, leading to significant training errors and poor convergence performance. To address this, researchers have employed intelligent algorithms to optimize ELM [31,32,33,34]. For instance, Wang M J et al. proposed the GWO-KELM model to enhance prediction performance [35], while Zeng N et al. introduced the SDPSO-ELM model, which outperformed RBFNN in experimental validation [36]. Q Sun et al. developed a PSO optimization algorithm to adaptively tune ELM parameters [37], and Liu Z et al. proposed the ERT-ELM model, which mitigates error accumulation in multi-step time-series prediction [38]. Despite these advances, ELM’s performance remains suboptimal, and as real-world optimization problems grow increasingly complex, the performance of swarm intelligence algorithms declines significantly. Shengwei Fu et al. introduced a novel metaheuristic algorithm, the Red-billed Blue Magpie Optimizer (RBMO), for engineering design problems, with its superior performance validated through extensive experiments [39].

Based on the above analysis, this paper proposes a new sensor-based tracking method that leverages the RBMO intelligent algorithm to synchronously optimize Improved Sequential Variational Mode Decomposition (ISVMD) and ELM, enhancing both signal decomposition and trajectory prediction. Specifically, the RBMO-optimized ISVMD decomposes and reconstructs target sequence features to generate the predictor’s input feature set, which the RBMO-optimized ELM then uses to predict the target’s trajectory. In simulated scenarios, the experimental results demonstrate that the RBMO-ISVMD-ELM algorithm effectively handles target information under complex random interference compared with other decomposition and unoptimized prediction algorithms, while accurately predicting trajectory trends. The main contributions of this study are as follows:

(1) With the ISVMD, the input feature set of a new predictor is constructed on the data–time axis. The algorithm is used to decompose complex signals efficiently and the best decomposition effect is obtained by statistical analysis. Compared to the existing VMD algorithm, this algorithm improves the decomposition accuracy and efficiency, which avoids the impact of abnormal data.
(2) With the help of the RBMO algorithm, a new predicting structure based on the ELM algorithm is designed for the overall system. The RBMO-ELM algorithm is directly used as a predictor, and the optimized hidden layer function and activation function are used to output the optimal prediction set. Compared with the traditional prediction algorithm, the algorithm improves the prediction accuracy and processing efficiency.
(3) The iterative operation process is grouped according to the data input time, and each data set is decomposed by the improved VMD algorithm and continuously passed to the predictor. In the prediction method, the RBMO algorithm is used to optimize the parameters of ISVMD and ELM algorithms simultaneously, and the prediction method performs self-learning and self-training in each iteration. This makes full use of the advantages of the new prediction method and realizes the characteristics of parallel computing.

The remainder of this paper is organized as follows: Section 2 describes the formation of the problem, the model of the detection space, and the noise interference. Section 3 describes the proposed RBMO-ISVMD-ELM method. Section 4 describes the experimental content. Finally, Section 5 gives conclusions and future research directions.

## 2. Preliminaries

In this section, we first describe a representative scenario in which millimeter-wave sensors mounted on an Unmanned Aerial Vehicle (UAV) are employed to track maneuvering targets within complex environments. Following the establishment of this scenario, we define the detection range space for the UAV and formulate a mathematical model for the signal reception process of the UAV. This model accounts for interference noise arising from both multi-path effects and ambient noise sources.

### 2.1. Problem Formulation

**Figure 1:**
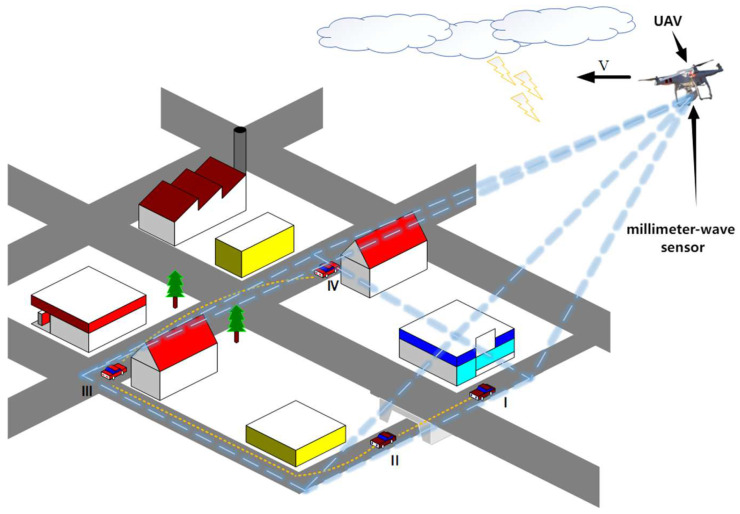
Tracking of maneuvering target by UAV in complex environment.

The experimental setup shown in Figure 1 features a variety of random environmental disturbances. These include phase shifts in received signals caused by multi-path effects, energy loss due to signal passage through obstacles, and signal distortions scattered within the natural environment. For simplicity, we assume a uniform type of interference throughout the study. The moving detection target operates within the UAV’s detection space. Specifically, the UAV’s sensor captures echo signals from this space, which are then processed by an onboard prediction system to predict the future path of the ground maneuvering target. Since time-varying environmental noise may mask the target’s dominant frequency signal, we will account for this in actual experiments.

We expect the prediction system to fully capture the signal characteristics in this environment, quickly decompose the signal, and enable rapid target tracking. Additionally, we require the maneuvering target to remain active within the detection space and be capable of moving to any position in the detection space.

### 2.2. Detection Space

**Figure 2:**
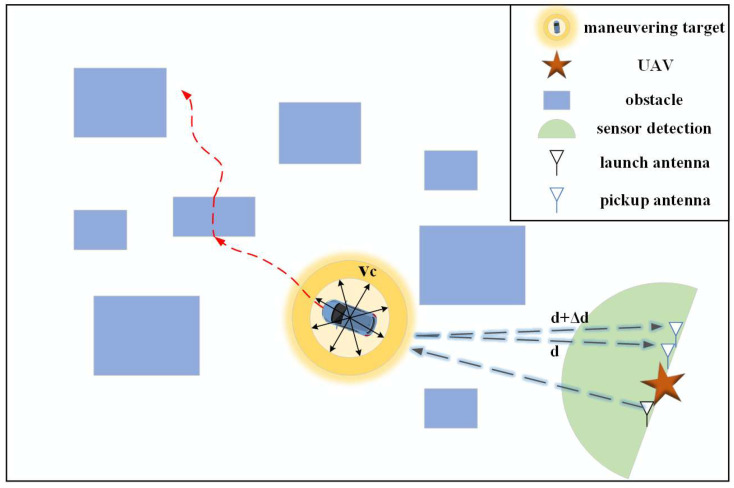
A schematic diagram of UAV detection space.

The detection space of the millimeter-wave sensor encompasses environmental perception data, state information of static or dynamic objects, and relative information. Specifically, environmental perception data primarily consists of random interference at varying frequencies, which mixes with target state information during sensor collection. The state information of static and dynamic objects includes relative distance and angle, derived from phase extraction and transformation. A schematic of the millimeter-wave sensor detection space is presented in Figure 2.

**Figure 3:**
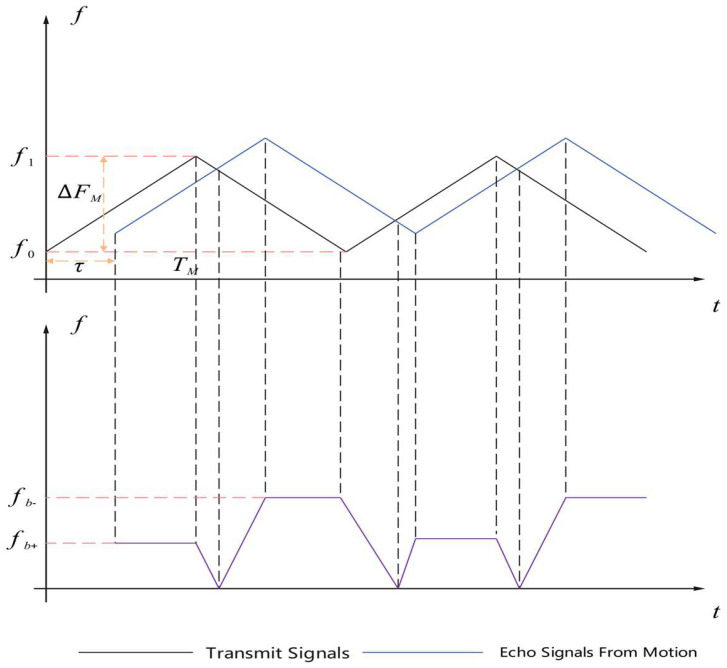
The distance measurement principle of triangular wave linear frequency modulation ranging.

To facilitate the processing of the trajectory predictor, it is necessary to preprocess the signals received in the detection space to calculate the relative distance and relative velocity of the target. We use millimeter-wave sensors mounted on the UAV as detectors to transmit signals and receive echo signals. The ranging principle of triangular wave linear frequency modulation in millimeter-wave sensors is shown in Figure 3.

**Assumption** **1.** *The sensor uses a triangular wave linear frequency modulation signal as the transmitting signal, which can be expressed as*

(1) $$S_{t}(t)=A_{t}\cos\left[2\pi \left(f_{c}t+kt^{2}/2\right)+\phi \right]$$

*where* t *is the time when the signal is transmitted,* $A_{t}$ *is the amplitude of the transmitted signal, k is the signal modulation rate,* ϕ *is the initial phase of the transmitted signal,* $f_{c}$ *is the center frequency of the signal, and* $T_{M}$ *is the modulation period,* $0\leq t\leq T_{M}/2$.

**Assumption** **2.** *The radial velocity of the sensor and the target is* v*, the radial distance is r, and the echo signal delayτis expressed as*

(2) $$\tau =\frac{2(r-vt)}{c}$$

Simplify the above equation as

(3) $$\tau =\tau_{0}-\Gamma t$$

where c denotes the speed of sound at room temperature, $\Gamma =\frac{2v}{c}$.

From **Assumption 1** and **Assumption 2**, the up-swept frequency band signal of the echo can be expressed as

(4) $$S_{r+}(t)=A_{t}k_{r}\cos\left\{2\pi \left[f_{c}(t-\tau )+k{\left(t-\tau \right)}^{2}/2\right]+\phi +\theta \right\}$$

where θ is the phase shift of the target echo signal and $k_{r}$ is the signal attenuation constant.

The target echo signal is mixed with the local oscillator signal, and the difference frequency signal of the up-swept band $S_{b+}(t)$ is expressed as

(5) $$S_{b+}(t)=\frac{k_{r}A_{0}^{2}}{2}\cos\left\{2\pi \left((1+\Gamma )k\tau_{0}-f_{d}\right)t-\left(2k\Gamma +k\Gamma^{2}\right)t^{2}+\left(f_{c}-\frac{k\tau_{0}}{2}\right)\tau_{0}-\frac{k\tau_{0}^{2}}{2}-\phi_{0}\right\}$$

Thus, the center frequency of the difference frequency signal $f_{b+}$ is

(6) $$f_{b+}=(1+\Gamma )k\tau_{0}-f_{d}$$

Similarly, the center frequency of the difference frequency signal of the down-swept frequency band can be obtained, as follows:

(7) $$\left\{\begin{array}{l} f_{b+}=k\tau_{0}-f_{d}=\frac{4R\Delta F_{M}}{T_{M}c}-f_{d}\\ f_{b-}=k\tau_{0}+f_{d}=\frac{4R\Delta F_{M}}{T_{M}c}+f_{d} \end{array}\right.$$

where $\Delta F_{M}$ is the frequency modulation bandwidth, $f_{d}$ is the Doppler shift, and its value is expressed as $\frac{f_{b-}-f_{b+}}{2}$.

The relative distance R and relative velocity v between the sensor and the target can be obtained by combining the above formulas, which are expressed as follows:

(8) $$\left\{\begin{array}{l} R=\frac{\left(f_{b+}+f_{b-}\right)T_{M}c}{8\Delta F_{M}}\\ v=\frac{\varepsilon }{2}f_{d}=\frac{\varepsilon }{4}\left(f_{b-}-f_{b+}\right) \end{array}\right.$$

where ε is the wavelength of the electromagnetic wave.

**Remark** **1.** *In order to avoid errors, it is necessary to use the low-pass filter and FFT technology to process the echo signal when the relative displacement and velocity measurement method of the above target are used. Moreover, we believe that using only improved VMD or separate phase analysis and conversion for signal separation during the UAV predictor solving process will limit their effectiveness. Therefore, the combination of these two methods is considered to ensure that the constructed input feature set has reliability and input optimization.*

### 2.3. Noise Interference

In a real environment, the signals received by the receiving antenna of the Radio Frequency (RF) front end contain certain interference signals, which can be expressed mathematically as follows:

(9) $$n_{\mathrm{multipath}\;}(t)=\sum_{i=1}^{M}a_{i}y\left(t-\tau_{i}\right)$$

Equation (9) denotes the multi-path effect noise, where *M* is the number of multi-paths, $a_{i}$ is the attenuation coefficient of the *i*-th path, and $\tau_{i}$ is the delay of the *i*-th path.

(10) $$n_{\mathrm{clutter}\;}(t)\sim N\left(0,\sigma^{2}\right)$$

Environmental noise can be regarded as a stationary generalized random process with a mean of zero and a variance of $\sigma^{2}$. After adding noise, the signal received by the sensor can be expressed as follows:

(11) $$f_{\mathbb{R}}=f(t)+n_{\mathrm{multipath}\;}(t)+n_{\mathrm{clutter}\;}(t)$$

## 3. Methods

This section initially elaborates on the enhancements made to the Sequential Variational Mode Decomposition (SVMD) algorithm. These improvements are designed to more effectively separate noise from valid target echo signals in received data, enabling its application in subsequent sequence prediction algorithms. Furthermore, this section explains the operational framework of the Extreme Learning Machine (ELM), detailing the process from signal input and activation function application to the generation of predictive outputs. The discussion then introduces the Red-billed Blue Magpie Optimization (RBMO) algorithm, which simultaneously optimizes both the ISVMD and ELM components. Finally, the complete algorithmic structure is established by integrating these methodologies.

### 3.1. ISVMD

The traditional SVMD method facilitates effective signal reconstruction by decomposing the input signal into multiple Intrinsic Mode Functions (IMFs), each characterized by a distinct center frequency. The optimized objective of this algorithm is to minimize the bandwidth of each mode, ensuring the compactness of the decomposition, while adhering to the constraint that the sum of all modes equals the original signal [40]. However, the decomposition accuracy of SVMD^1^ is constrained by its reliance on a single L2 norm penalty term, which may introduce signal reconstruction errors or mode mixing. To overcome this limitation, this section introduces a hybrid penalty term combining both L1 and L2 norms to enhance both the smoothness and sparsity of the signal simultaneously. The objective function for the ISVMD is defined as follows:

(12) $$\min_{f}\left\{{\left\|f-f_{\mathbb{R}}\right\|}_{2}^{2}+\alpha {\left\|\partial_{t}f\right\|}_{2}^{2}+\beta {\left\|\partial_{t}f\right\|}_{1}\right\}$$

where ${\left\|f-f_{\mathbb{R}}\right\|}_{2}^{2}$ denotes the distance between f and $f_{\mathbb{R}}$, $\alpha {\left\|\partial_{t}f\right\|}_{2}^{2}$ and $\beta {\left\|\partial_{t}f\right\|}_{1}$ denote the gradient-based L2 and L1 norms, respectively. The constrained variational equation for ISVMD is formulated as

(13) $$\begin{array}{l} \min_{\left\{v_{k}\right\},\left\{\omega_{k}\right\}}\left\{\alpha \sum_{k}{\left\|\partial_{t}\left[\left(\delta (t)+\frac{j}{\pi t}\right)*v_{k}(t)\right]e^{-j\omega_{k_{t}}}\right\|}_{2}^{2}+\beta {\sum_{k}\left\|\partial_{t}\left[\left(\delta (t)+\frac{j}{\pi t}\right)*v_{k}(t)\right]\right\|}_{1}\right\}\\ s.t.\;\sum_{k}v_{k}=f \end{array}$$

where $\left\{v_{k}\right\}$ is the intrinsic mode function of k single components, $\left\{\omega_{k}\right\}$ is the center frequency of each component, α is the weight parameter controlling L2 norm, β is the weight parameter controlling L1 norm, and δ(t) is the Dirac function. From the equation above, it is evident that the quadratic penalty term yields a global minimum. To integrate the constraints into the optimization framework, a Lagrange multiplier λ(t) is incorporated in Equation (13), converting the constrained optimization problem into an equivalent unconstrained form. The resulting Lagrange function is expressed as

(14) $$\begin{array}{l} L\left(\left\{v_{k}\right\},\left\{\omega_{k}\right\},\lambda \right)=\alpha \sum_{k}{\left\|\partial_{t}\left[\left(\delta (t)+\frac{j}{\pi t}\right)*v_{k}(t)\right]e^{-j\omega_{k}t}\right\|}_{2}^{2}\\ +\beta \sum_{k}{\left\|\partial_{t}\left[\left(\delta (t)+\frac{j}{\pi t}\right)*v_{k}(t)\right]e^{-j\omega_{k}t}\right\|}_{1}\\ +{\left\|f(t)-\sum_{k}v_{k}(t)\right\|}_{2}^{2}+\langle \lambda (t),f(t)-\sum_{k}v_{k}(t)\rangle \end{array}$$

Given the complexity of convolution and derivative operations in the time domain, the computation of Equation (14) is simplified by applying the Fourier transform, shifting the representation from the time domain to the frequency domain. Accounting for the derivative terms and frequency shifts of the modes, the Fourier transform of the equation is given by:

(15) $$F\left\{\partial_{t}\left[\left(\delta (t)+\frac{j}{\pi t}\right)*v_{k}(t)\right]e^{-j\omega_{k}t}\right\}=j\omega \left[1+\mathrm{sgn}\left(\omega +\omega_{k}\right)\right]{\hat{v}}_{k}\left(\omega +\omega_{k}\right)$$

where ${\hat{v}}_{k}\left(\omega +\omega_{k}\right)$ denotes the frequency shift of the mode in the frequency domain. Following this transformation, Equation (15) is reformulated in the frequency domain as

(16) $$\begin{array}{l} L\left(\left\{{\hat{v}}_{k}\right\},\left\{\omega_{k}\right\},\hat{\lambda }\right)=\alpha \sum_{k}\int_{-\infty }^{\infty }{\left|j\omega \left[1+\mathrm{sgn}\left(\omega +\omega_{k}\right)\right]{\hat{v}}_{k}\left(\omega +\omega_{k}\right)\right|}^{2}d\omega \\ +\beta \sum_{k}\int_{-\infty }^{\infty }\left|j\omega \left[1+\mathrm{sgn}\left(\omega +\omega_{k}\right)\right]{\hat{v}}_{k}\left(\omega +\omega_{k}\right)\right|d\omega +\int_{-\infty }^{\infty }{\left|\hat{f}(\omega )-\sum_{k}{\hat{v}}_{k}(\omega )\right|}^{2}d\omega \\ +\int_{-\infty }^{\infty }\hat{\lambda }(\omega )\left(\hat{f}(\omega )-\sum_{k}{\hat{v}}_{k}(\omega )\right)d\omega \end{array}$$

To ensure both the convergence and computational efficiency of Equation (16), the Alternating Direction Method of Multipliers (ADMM) is employed to solve the formulation [36]. For the update of the modes, a subproblem is constructed to minimize the Lagrange function with respect to a single variable, expressed as

(17) $${\hat{v}}_{k}^{n+1}(\omega )=\arg\min_{{\hat{v}}_{k}(\omega )}\left\{\begin{array}{l} \alpha \int_{0}^{\infty }4{\left(\omega -\omega_{k}\right)}^{2}{\left|{\hat{v}}_{k}(\omega )\right|}^{2}d\omega +\beta \int_{0}^{\infty }2\left|j\left(\omega -\omega_{k}\right){\hat{v}}_{k}(\omega )\right|d\omega \\ +\int_{-\infty }^{\infty }{\left|\hat{f}(\omega )-\sum_{i}{\hat{v}}_{i}(\omega )+\frac{\hat{\lambda }(\omega )}{2}\right|}^{2}d\omega \end{array}\right\}$$

When $\omega -\omega_{k}>0$, the integral is evaluated over the positive frequency range, simplifying the norm computation. Solving Equation (17) yields the update formula for $v_{k}$, which is given by

(18) $${\hat{v}}_{k}^{n+1}(\omega )=\frac{\hat{f}(\omega )-\sum_{i\neq k}{\hat{v}}_{i}(\omega )+\frac{\hat{\lambda }(\omega )}{2}}{1+4\alpha {\left(\omega -\omega_{k}\right)}^{2}+\beta j\left(\omega -\omega_{k}\right)}$$

Similarly, a subproblem for $\omega_{k}$ is formulated to adjust $\omega_{k}$ to minimize the mode’s bandwidth, expressed as

(19) $$\omega_{k}^{n+1}=\frac{\int_{0}^{\infty }\omega {\left|{\hat{v}}_{k}(\omega )\right|}^{2}d\omega }{\int_{0}^{\infty }{\left|{\hat{v}}_{k}(\omega )\right|}^{2}d\omega }$$

Detailed derivation of Equations (18) and (19) is provided in Appendix A.

Finally, the Lagrange multiplier is updated to ensure progressive alignment with the constraint condition. Following the ADMM approach, the update formula incorporates a step size μ for adjustment, ensuring compliance with the constraint, and is expressed as

(20) $${\hat{\lambda }}^{n+1}(\omega )={\hat{\lambda }}^{n}(\omega )+\mu \left(\hat{f}(\omega )-\sum_{k}{\hat{v}}_{k}^{n+1}(\omega )\right)$$

This update formula leverages the residual term to refine *y*, thereby incrementally reducing the signal reconstruction error with each iteration. Through successive iterative updates to the ${\hat{v}}_{k}$, $\omega_{k}$, and $\hat{\lambda }$, Equation (14) converges to an optimal solution that satisfies both the optimization objective and the imposed constraints.

### 3.2. ELM

The ELM neural network demonstrates remarkable competence in processing non-linear data, effectively capturing the dynamic characteristics of input signals—a critical advantage for predicting trajectories with complex motion patterns. By incorporating a self-connection mechanism, ELM leverages historical information to forecast future data points, making it particularly suited for time-series prediction tasks like trajectory analysis. Notably, the hidden layer weights of ELM are randomly initialized and require no adjustment during training, a design that significantly accelerates the training process while preserving strong generalization performance. Compared to traditional neural networks, ELM reduces the number of tunable parameters, thereby minimizing overfitting risks and enhancing model stability.

**Figure 4:**
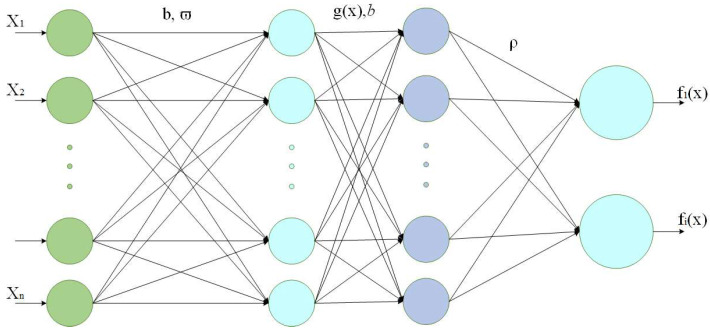
ELM network framework.

Structurally, ELM comprises input, output, and hidden layers. Its robust learning capability and simple algorithmic architecture make it ideal for solving complex non-linear function problems. The hidden layer serves as the core component: the ELM algorithm solves hidden layer biases by computing the generalized inverse matrix, iteratively refining the solution to gradually reduce errors. The framework of the ELM network is illustrated in Figure 4.

Assuming that the number of nodes in the hidden layer is L, the input weight $\varpi_{i}$ and the hidden layer offset $b_{i}$ are randomly generated. To introduce non-linear transformations in ELM and enhance the model’s expressive ability, we choose the sigmoid function as the activation function g(x), and the hidden layer output matrix is calculated as follows:

(21) $$H=\sum_{i=1}^{L}g\left(\varpi_{i}x_{j}+b_{i}\right)$$

Therefore, the output weight special solution ρ and the global optimal output matrix can be calculated. The formula is as follows:

(22) $$\rho =H^{+}T$$

According to the algorithm structure characteristics of the ELM, it can be used as a predictor to predict future estimation more efficiently and accurately. However, due to the manual intervention of the parameters of the traditional ELM, the output result is not ideal. Based on the traditional ELM, this paper integrates the RBMO optimization algorithm to adapt the parameters selected by manual experience, which greatly improves the efficiency and prediction accuracy of the algorithm. This will be proved in subsequent experimental verification.

### 3.3. The Optimized Algorithm of RBMO

As a signal decomposition algorithm, the VMD algorithm is greatly affected by the parameters during operation, and the parameters set by artificial experience cannot achieve the best results [41], where k and a are the key factors that determine the quality of signal decomposition. When k and a are too small, the decomposed signal is prone to aliasing. When the two values are too large, it is prone to overdecomposition. As a predictor, although the ELM has excellent prediction performance, the number of hidden internal layer nodes and activation functions still need to be adjusted by manual experience, which leads to inappropriate parameter selection greatly affecting the performance and efficiency of the predictor. When the number of nodes is too large, the algorithm takes up a lot of computing resources, which is not suitable for the real-time performance of target trajectory prediction, and there will be an overfitting phenomenon. When the number of nodes is too small, it will lead to poor prediction performance. Improper parameter selection of the activation function in the ELM algorithm will lead to poor robustness and non-convergence of the model.

In this section, we use the MAE in the ELM predictor as the fitness function and use the RBMO optimization algorithm to adaptively select the number of hidden layers and the parameters of the activation function and finally obtain the optimal weights and thresholds. At the same time, we introduce the permutation entropy and the decomposition balance factor to adaptively adjust the fitness function of the RMBO algorithm and then optimize the three parameters of the ISVMD, namely, the number of optimized components, the penalty functions α and β. Specifically, RBMO adopts a single-objective optimization strategy, where the joint objective function design uses the Mean Absolute Error (MAE) of the ELM predictor as the fitness function [41], and high-quality decomposition features are generated as inputs for the ELM, thereby indirectly improving prediction accuracy. Auxiliary indicators such as Permutation Entropy (PE) which measure the complexity of the modes and Decomposition Balance Factor (DBF) which ensures the balance of energy distribution among modes are used to constrain the decomposition quality. Although RBMO does not explicitly adopt multi-objective optimization, an implicit balance between the decomposition quality and the prediction accuracy of ELM is achieved through MAE. The mechanism by which RBMO jointly optimizes ISVMD and ELM is detailed in Algorithm 1.
**Algorithm 1** ISVMD-ELM-based RBMO optimization1.Combining RBMO with ISVMD and ELM2.Initialize ISVMD relate parameters3.Initialize ELM network parameters4.**for** each episode **do**5.  Initialize an observation queue and observation variables $f_{0}(x)$:6.  **obs_a** = queue ([**0**, **0**, …, **0**, $f_{0}(x)$])7.  **for** step t = 0, T−1 **do**8.   κ,α←RBMO;$\varpi_{i},b_{i}\leftarrow RBMO$9.    $\left\{v_{k}\right\},\left\{\omega_{k}\right\}\leftarrow L\left(\left\{v_{k}\right\},\left\{\omega_{k}\right\},\lambda \right)$;$\min f(x)+h(y)\leftarrow \left(x^{*},y^{*},z^{*}\right)$10.    ${\hat{v}}_{k}^{n+1}(\omega )\leftarrow \underset{{\hat{k}}_{k}(\omega )}{\arg\min}\{s(\omega )\}$;${\hat{\omega }}_{k}^{n+1}(\omega )\leftarrow \underset{{\hat{v}}_{k}(\omega )}{\arg\min}\left\{{\hat{v}}_{k}(\omega )|d\omega \right\}$11.    $E_{i}^{j}\leftarrow randN{\left\{\left(x_{i},t_{i}\right)\right\}}_{i=1}^{N}$;$\varepsilon_{j}\leftarrow \sum \varpi_{j}(i)$12.    $H\leftarrow \sum_{i=1}^{L}g\left(\varpi_{i}x_{j}+b_{i}\right)$;$\rho \leftarrow H^{+}T$13.    
$f(x)\leftarrow queue(f_{t}(x)|\varpi_{i},b_{i})$
14.    Add $f_{t+1}(x)$ to the observation queue:15.   **obs_a** = **obs_a**.append($f_{t+1}(x)$)16.   
**end for**
17.**end for**

**Remark** **2.** *A high PE value indicates overly complex modes, while a low DBF value reflects uneven energy distribution among modes, both of which affect MAE and are adjusted during the optimization process. Furthermore, RBMO’s data-driven adaptive optimization reduces the reliance on manual parameter tuning for ISVMD and ELM, enhancing the model’s robustness and performance under different signal characteristics.*

### 3.4. Algorithm Structure

**Figure 5:**
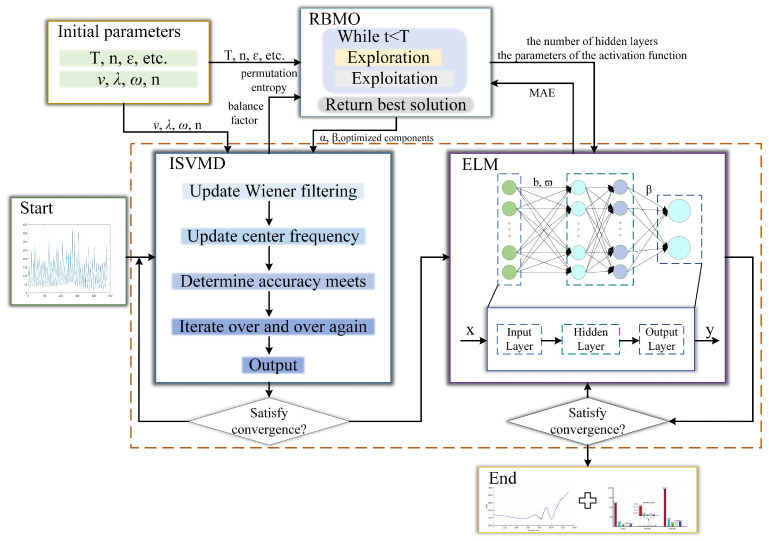
Overall prediction system structure.

By optimizing and enhancing both the structure and the input end of the predictor, we have achieved improvements in the speed and accuracy of the prediction system when applied to the prediction of target trajectories. The prediction method designed in this paper for predicting the trajectory of ground maneuvering targets can be fully applied to complex environments. The prediction framework is as follows: firstly, the target motion signal of complex multi-frequency interference is precollected and decomposed and reconstructed by the VMD algorithm. RBMO is used to optimize VMD parameters to ensure that the decomposed signals do not show aliasing. Subsequently, the RBMO-optimized ELM algorithm is used to solve the problem of low prediction performance of traditional algorithms. The architecture of double threshold and repeated iteration is used to ensure that the predicted results are closer to the target motion state. The overall prediction system structure is shown in Figure 5.

## 4. Experiments and Results

This section aims to validate the algorithm’s specific performance. The algorithm’s signal decomposition accuracy and adaptability demonstrate its stability in more complex, highly interfered environments. The trajectory prediction algorithm’s tracking accuracy reflects its precision and stability in scenarios where targets may be randomly lost. Ultimately, through comprehensive evaluations and cross-algorithm comparisons, we verify the superiority of our method.

**Figure 6:**
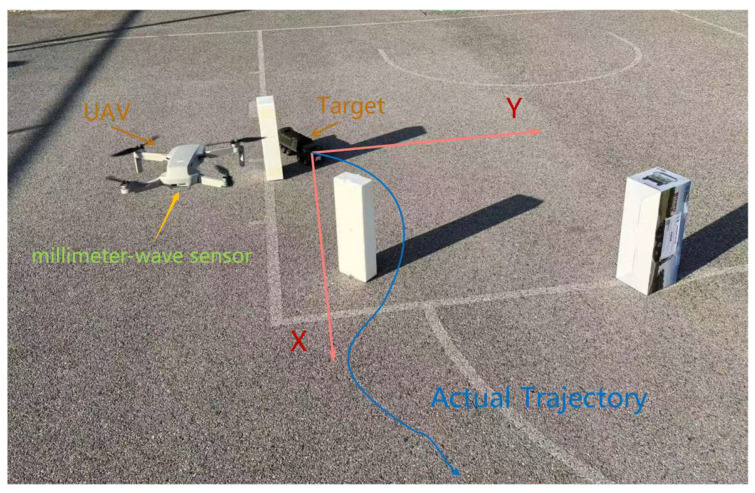
The actual experimental scene layout diagram.

As illustrated in Figure 6, we established a real experimental environment: a drone equipped with a millimeter-wave radar platform was used to detect the positions of moving ground vehicles. Random obstacles were set up in the experiment to partially obscure the vehicles’ trajectories. After data collection, the drone transmitted the data to a ground station for algorithmic processing and analysis.

### 4.1. Experiment Setting

In this experimental section, we design two experiments to comprehensively validate the algorithm. Firstly, we generate a simulated signal using three modulated cosine signals (50 Hz, 100 Hz, and 150 Hz) combined with white noise at a Signal-to-Noise Ratio (SNR) of 10 dB to validate the algorithm’s decomposition performance on mixed-frequency signals. Subsequently, the algorithm is encapsulated and integrated with a millimeter-wave radar module mounted on a UAV, serving as the input sensor for signal acquisition. The selected module is compact, easy to deploy, exhibits stable signal transmission power, and offers adjustable signal characteristics. A scenario with obstacles was established under conditions featuring wind noise and realistic telemetry noise. A remote-controlled car was chosen as the detection target, and the signal data received by the millimeter-wave module on the UAV were used to evaluate the decomposition accuracy and adaptability of the proposed algorithm. The prediction results of the algorithm were compared with those obtained through a comprehensive evaluation method.

### 4.2. Experiment Parameters and Evaluating Indicator

Under the above experimental conditions, the specific parameter settings of the millimeter-wave sensor and algorithms are shown in Table 1 and Table 2.

We used common indicators such as MAE, MSE, and RMSE to evaluate the overall prediction performance of the algorithm. The indicators are defined as follows:

(23) $$MAE=\frac{1}{n}\sum_{i=1}^{n}\left|x_{i}-{\hat{x}}_{i}\right|$$

(24) $$MSE=\frac{1}{n}\sum_{i=1}^{n}{\left(x_{i}-{\hat{x}}_{i}\right)}^{2}$$

(25) $$\mathrm{RMSE}=\sqrt{\frac{1}{n}\sum_{i=1}^{n}{\left({\hat{x}}_{i}-x_{i}\right)}^{2}}$$

where ${\hat{x}}_{i}$ is the *i*-th predicted value of the model; $x_{i}$ is the *i*-th real goal trajectory value. MAE is used to measure the average absolute deviation from the predicted target position. MSE amplifies the feedback on errors due to its squared term. RMSE provides the standard deviation of the prediction error, reflecting the algorithm’s sensitivity to errors. The above three metrics will reflect the Euclidean distance error between the predicted and actual results for the target position.

### 4.3. Performance Evaluation of Algorithms

#### 4.3.1. Performance Comparison of Decomposition Algorithms

**Figure 7:**
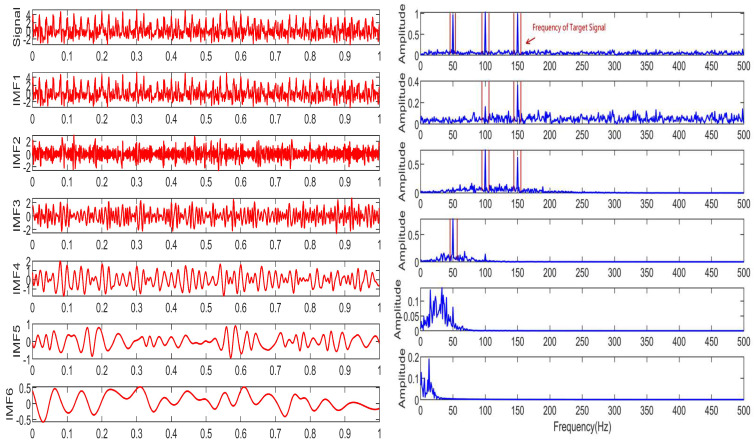
The decomposition result of CCEMD algorithm.

**Figure 8:**
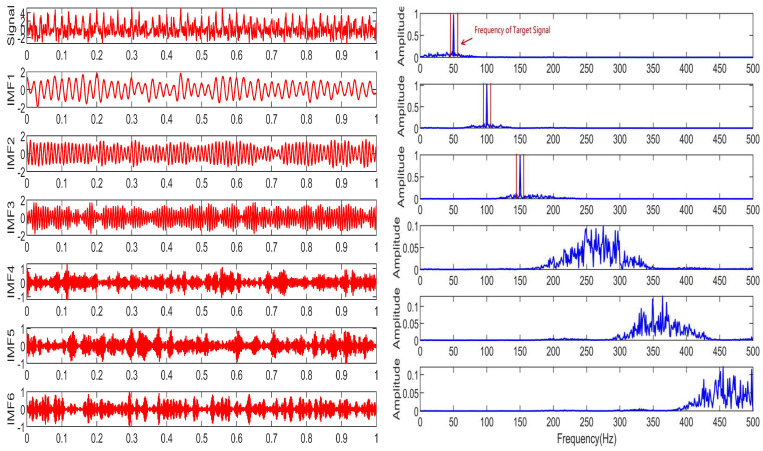
The decomposition result of VMD algorithm.

**Figure 9:**
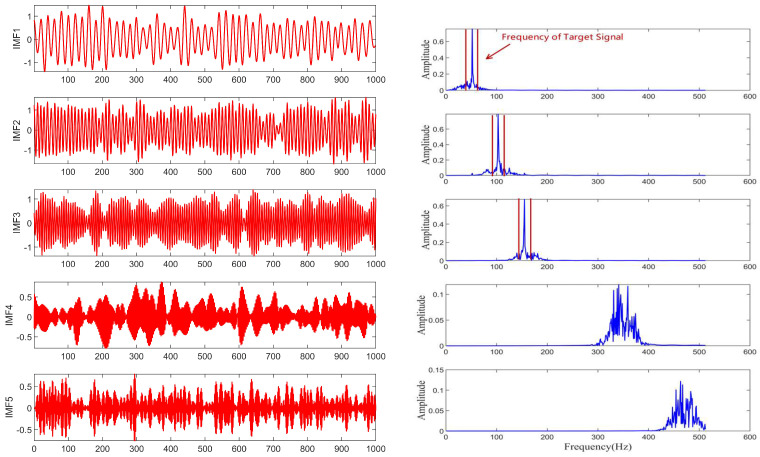
The decomposition result of RBMO-ISVMD algorithm.

The performance of the signal decomposition algorithm depends on the efficient and accurate decomposition results of the mixed frequency signal. To more accurately verify the superior performance of the proposed algorithm, this paper uses three algorithms for simulation comparison, namely CCEMD, VMD, and RBMO-ISVMD. The decomposition effects of the three algorithms are shown in Figure 7, Figure 8 and Figure 9.

From Figure 7, Figure 8 and Figure 9, it is evident that the CCEMD algorithm still exhibits frequency aliasing after signal decomposition. Although the VMD algorithm approaches the decomposition effect of the algorithm proposed in this paper, this is because the parameter k in the VMD algorithm employs an optimal value derived from experience, whereas the algorithm proposed herein possesses inherent adaptability. When the set k-value fails to meet the optimal threshold, decomposition modes may become distorted or incomplete, leading to aliasing. By contrast, the signal decomposed by our algorithm clearly resolves three fundamental frequencies: 50 Hz, 100 Hz, and 150 Hz, demonstrating superior performance.

**Figure 10:**
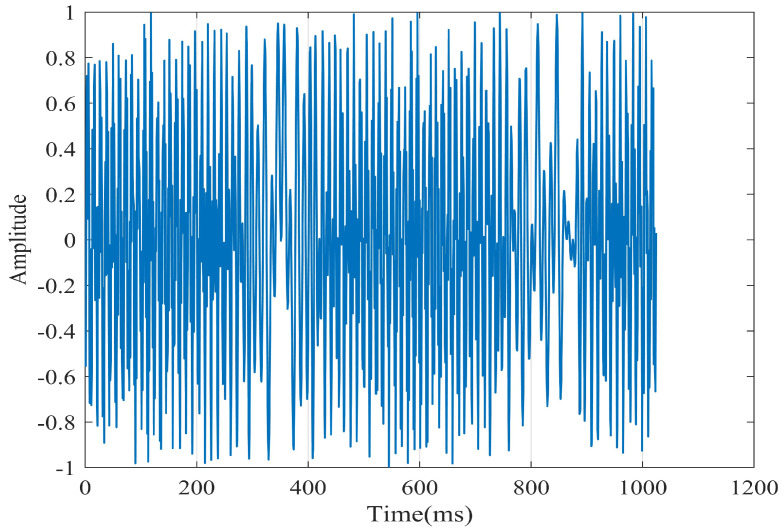
The difference in frequency signal diagram of the target at three meters.

**Figure 11:**
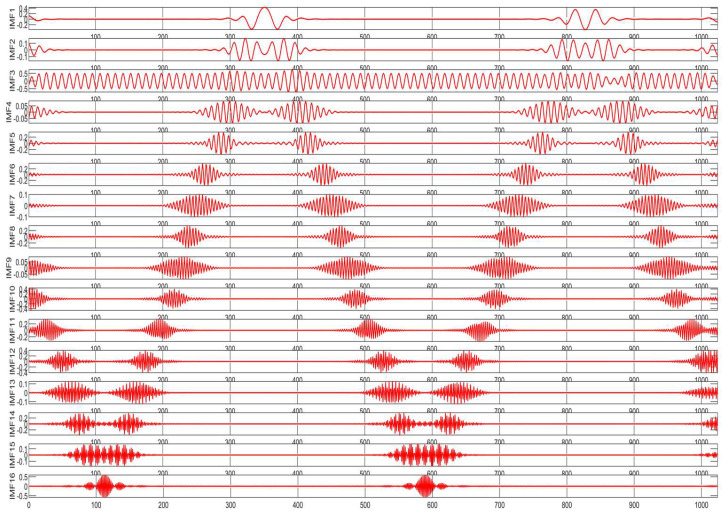
The time domain diagram processed by RBMO-ISVMD algorithm.

**Figure 12:**
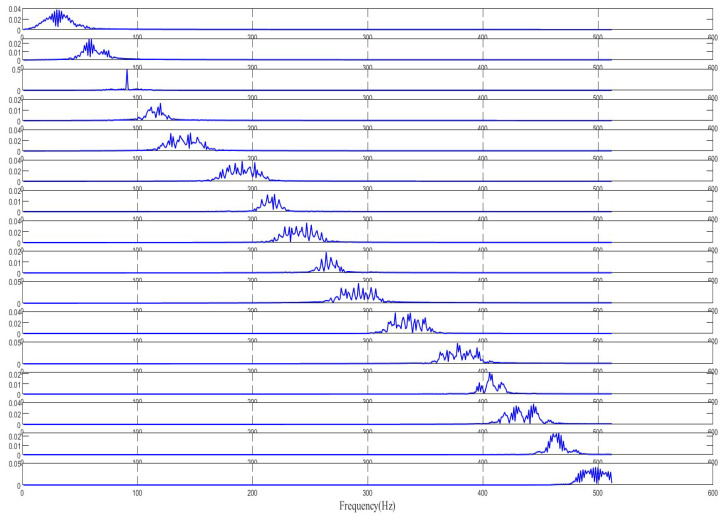
The spectrum diagram processed by RBMO-ISVMD algorithm.

**Figure 13:**
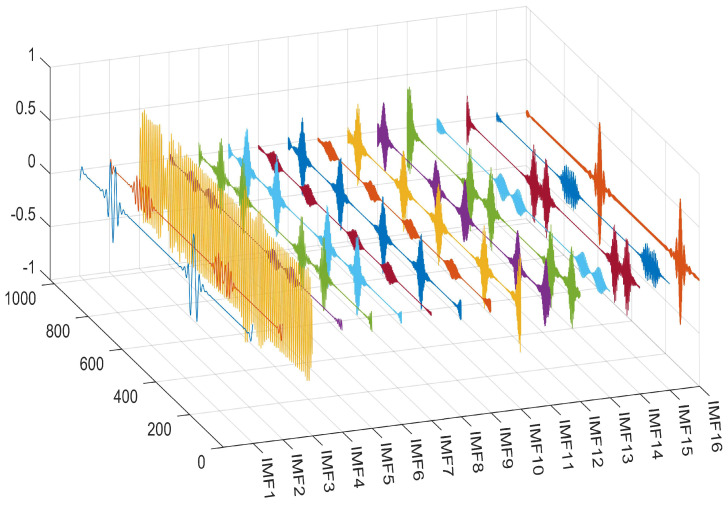
The schematic diagram of time domain and spectrum.

**Figure 14:**
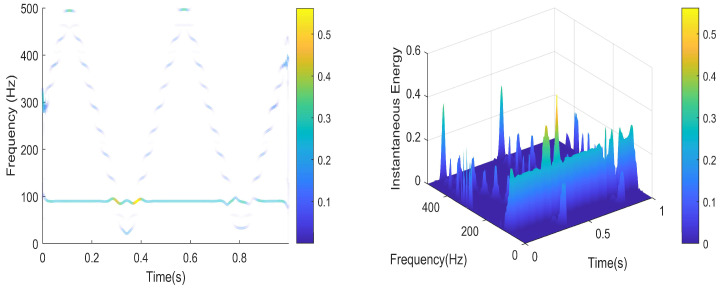
The schematic diagram of Hilbert spectrum and instantaneous energy.

**Figure 15:**
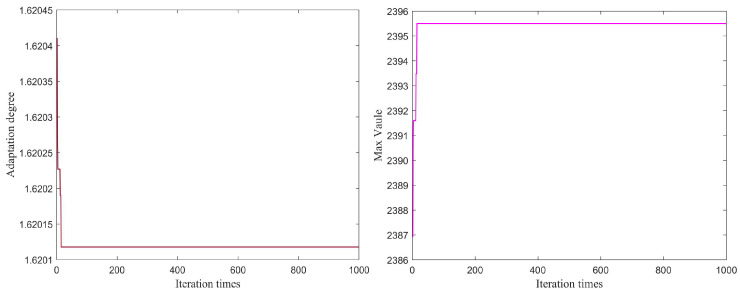
The schematic diagram of adaptation degree and max value.

To further validate the algorithm, we utilized an MMWCAS-RF-EVM four-chip cascaded millimeter-wave radar coupled with a data acquisition card to receive beat frequency signals from targets positioned 1 to 6 m from the sensor. The algorithm was employed to derive the target’s precise beat frequency, from which the target’s distance was calculated using the ranging formula. An illustrative example of processing the target beat frequency signal at a 3-m distance is provided in Figure 10, Figure 11, Figure 12, Figure 13, Figure 14 and Figure 15.

From Figure 10, Figure 11, Figure 12, Figure 13, Figure 14 and Figure 15, the proposed algorithm can completely adaptively decompose and reconstruct the signal modulus when dealing with analog signals containing random noise. IMF3 in Figure 11 is completely separated from other IMFs. From Figure 14, the reconstructed frequency value is 90.2 Hz. According to the ranging formula, the distance of the target can be calculated to be 3.0067 m, and the distance error with the set target is only 0.0067 m. From Figure 15, it can be observed that the fitness function gradually decreases with the increase in iterations, reaching its minimum and stabilizing after eight iterations. At this point, the optimal parameter values have been computed, demonstrating the rapid convergence of the proposed algorithm. The ISVMD algorithm, optimized by the RBMO algorithm, achieves superior decomposition accuracy and adaptability.

According to the above simulation method, we then analyze the target difference frequency signal from 1 to 6 m, and the total results are shown in Table 3.

**Remark** **3.** *Since the measurement error of the default angle in this paper can be ignored, only the measurement error of the target at different distance radii is compared when solving the target difference frequency signal continuously output by the millimeter-wave module.* *In addition, the angle information needs to be added when calculating the two-dimensional coordinate points of the target motion.*

**Figure 16:**
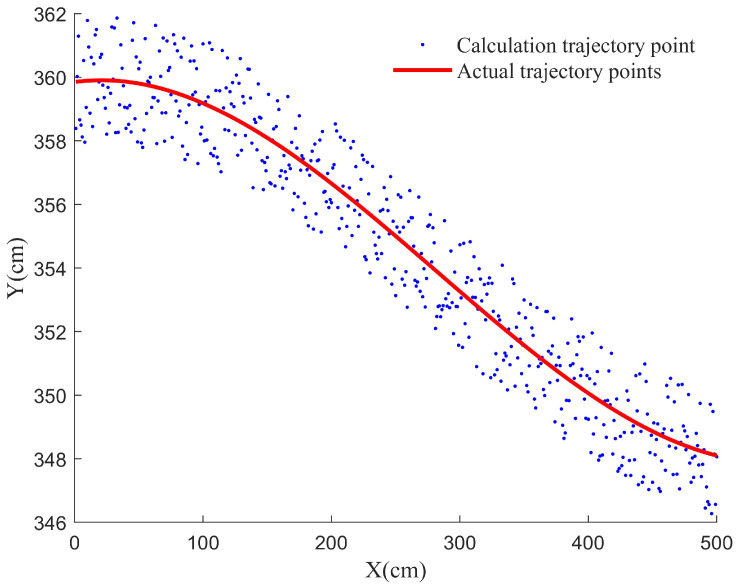
The diagram of the calculation and actual target trajectory points.

Based on the aforementioned experimental, we process the target difference frequency signal continuously transmitted by the millimeter-wave module in the UAV. The target distance information decomposed by the RBMO-ISVMD algorithm and the target angle information are calculated. We obtain the historical trajectory points of the target in the detection space. The average error between the calculated historical trajectory points and the actual target marker points is calculated, and the result is 0.02 m, which is almost close to the actual marker points. The calculation and actual target trajectory points are shown in Figure 16.

#### 4.3.2. Performance Comparison of Prediction Algorithms

Before implementing the prediction algorithm, we preprocess the set of target historical trajectories to serve as the input sequence for the prediction model. In this algorithm, an input sequence comprising 500 sample points is partitioned into 20 sample sets, of which 19 are for training and 1 for testing. With a sliding window size set to 2, the prediction algorithm executes training, derivation, and verification processes across the entire input sequence.

**Figure 17:**
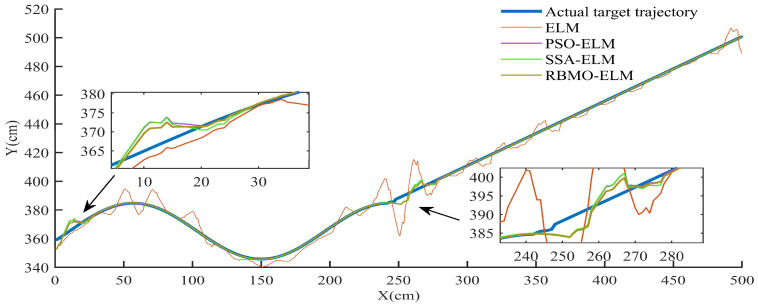
Comparison results of different prediction algorithms.

**Figure 18:**
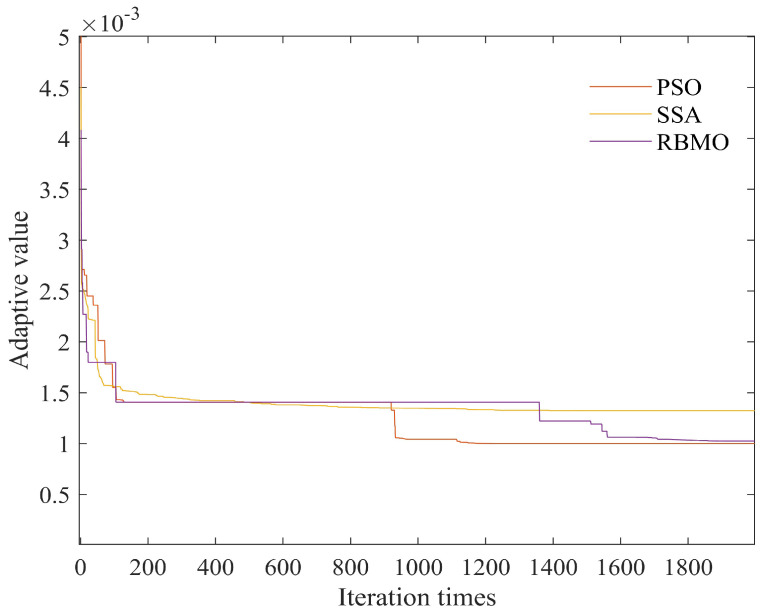
The schematic diagram of the adaptive value.

As derived from Section 4.3.1, the historical trajectory points of maneuvering targets within the detection space are obtained. To validate the proposed algorithm’s superiority, we compare it with alternative prediction methods: ELM, PSO-ELM, SSA-ELM, and RBMO-ELM. The comparative results are visualized in Figure 17 and Figure 18.

Figure 17 and Figure 18 illustrate the comparative prediction accuracy and performance of the four algorithms. Figure 15 highlights the prediction errors in target motion trajectory after individually forecasting the X and Y coordinates and then fusing the results. Initially, there is a significant prediction error due to the target’s deviation from its historical trajectory (Figure 17), but RBMO-ELM achieves the smallest error and rapidly adapts to these changes compared to the other algorithms. Similarly, in the second phase, as the target transitions from a curved motion pattern to a linear one, the algorithms also exhibit prediction errors. In both scenarios involving changes in the target’s motion, RBMO-ELM demonstrates the best performance, followed by SSA-ELM and PSO-ELM, with the traditional ELM performing the worst.

Figure 18 displays the fitness function variation of the three optimization algorithms. Although RBMO does not converge as quickly as PSO, its final convergence result is superior, achieving the smallest and most stable value. Table 4 presents the performance metrics of four comparative algorithms in target trajectory prediction, including the ELM, PSO-ELM, SSA-ELM, and RBMO-ELM. The traditional ELM exhibits an MAE of 3.645 m, an MSE of 28.288 square meters, and an RMSE of 5.31865 m, indicating large and unstable prediction errors. In contrast, the PSO-ELM achieves an MAE of 0.380712 m, an MSE of 1.36262 square meters, and an RMSE of 1.16731 m, demonstrating that particle swarm optimization significantly enhances the prediction accuracy of the ELM. The SSA-ELM records an MAE of 0.512302 m, an MSE of 1.43197 square meters, and an RMSE of 1.19665 m, with performance slightly inferior to that of the PSO-ELM. The proposed RBMO-ELM algorithm yields an MAE of 0.392579 m, an MSE of 1.09496 square meters, and an RMSE of 1.04641 m. Although its MAE is slightly higher than that of the PSO-ELM, its MSE and RMSE outperform the other three algorithms. These results collectively indicate that the RBMO-ELM algorithm excels in comprehensive error control and stability, particularly in high-precision trajectory prediction in complex environments.

Among the four comparative algorithms, the training complexity of the unoptimized ELM primarily depends on the sample size and the number of hidden layer nodes, resulting in a relatively low computational load. The prediction phase requires only a single, simple matrix operation. In contrast, PSO-ELM, SSA-ELM, and RBMO-ELM introduce iterative optimization to adjust parameters, increasing the computational load during training, but their computational complexity in the prediction phase remains identical to that of the traditional ELM. The RBMO-ELM incurs a higher computational cost during training due to the simultaneous optimization of ISVMD and ELM parameters, as well as the handling of signal decomposition mode numbers, but its prediction phase maintains the same computational efficiency as the ELM, ensuring an efficient processing pipeline. Although the RBMO-ELM entails greater training overhead, it offers high efficiency during prediction, making it suitable for real-time applications. Compared to the other three algorithms, RBMO-ELM achieves superior prediction performance with comparable training costs (Table 4).

In summary, the method proposed in this paper has been well validated in static noise and single-target scenarios. Its excellent results are attributed to the algorithm’s adaptive decomposition and generalization capabilities. Furthermore, within this algorithm, ISVMD can adjust parameters through RBMO optimization to adapt to time-varying noise. By combining ISVMD with the optimized ELM, the method enables multi-channel signal decomposition and multi-target sequence prediction for multiple targets. In future work, we will validate the method in more complex scenarios.

## 5. Conclusions and Discussion

A new prediction method for millimeter-wave sensor tracking of maneuvering target trajectories (RBMO-ISVMD-ELM) is proposed. This algorithm employs an RBMO-optimized Improved Sequential Variational Mode Decomposition (ISVMD) as a signal decomposer to precisely dissect and reconstruct received signals, ensuring the accurate extraction of historical features from maneuvering targets. Leveraging an Extreme Learning Machine (ELM) as the predictor enhances trajectory forecasting accuracy, while the Red-billed Blue Magpie Optimizer (RBMO) fine-tunes critical parameters to optimize system adaptability and computational efficiency. Comparative simulations reveal that the proposed method outperforms existing algorithms, particularly in scenarios characterized by strong interference and intermittent signal loss. Experimental results further confirm that both the enhanced decomposition algorithm and optimized parameter tuning significantly elevate the overall performance of the predictive framework.

In the future, we will continue to optimize the sensor tracking system’s solution methods, evaluating the impact of incorporating different modes on tracking effectiveness and conducting research on multi-target synchronous tracking. These efforts will extend to practical deployment in real-world scenarios, such as integrating the system into UAVs for field testing, ensuring practicality by developing user-friendly features for operators, and addressing system resource requirements by optimizing computational demands to align with more UAV hardware constraints. The new prediction method will be applied to enhance the front-end design of the UAV control system, improving both performance and usability in UAVs.

## Figures and Tables

**Table 1:** The parameters of MMWCAS-RF-EVM four-chip cascade millimeter-wave sensor.

Experimental Parameters	Value
Initial frequency	77 GHz
Frequency modulation slope	32 MHz/μs
Chirped signal period	40 μs
Number of chirped signals	64
Frame length	100 ms

**Table 2:** The specific parameters of the algorithm.

	Experimental Parameters	Value
ISVMD	α range	[500,3000]
	β range	[0.1,1.0]
	Time-step of the ascent	0
	Tolerance	1 × 10^−6^
	Condition of convergence	4
ELM	Initial input layer	20
	Initial hidden layer	20
	Initial output layer	2
	Prediction horizon	5
	Activation functions	{sigmoid, tanh, ReLU}
RBMO	Upper limit of equilibrium parameters	1000
	Lower limit of equilibrium parameters	5000
	Maximum number of cycles	200
	Population size	30
	Exploration rate	0.7
	Cognitive factor	2.0
	Initialized loudness	1

**Table 3:** The total results of target distance solving.

Setting Distance of Target (m)	Distance Calculation Results (m)	Error (m)
1	1.0067	0.0067
2	2.0033	0.0033
3	3.0067	0.0067
4	4.02	0.02
5	5.0233	0.0233
6	6.03	0.03

**Table 4:** Performance indicators of different prediction algorithms.

	ELM	PSO-ELM	SSA-ELM	RBMO-ELM
MAE (m)	3.645	0.380712	0.512302	0.392579
MSE (m^2^)	28.288	1.36262	1.43197	1.09496
RMSE (m)	5.31865	1.16731	1.19665	1.04641

## Data Availability

The open-source code and data are released at https://github.com/xingying123/A-new-prediction-method-for-UAV.git (Established on 16 September 2014).

## Appendix A

The update formulas are constructed using ADMM, with the detailed derivation and the specific expression for the optimal solution of the equation using ADMM as follows:

(A1) $$\begin{array}{l} \min f(x)+h(y)\;\\ s.t.\;Ax+By+b=0 \end{array}$$

Then, $L_{v}(x,y,z)$ can be expressed as:

(A2) $$L_{v}(x,y,z)=f(x)+h(y)+z^{T}(Ax+By+b)+\frac{v}{2}{\left\|A,x,+,B,y,+,b\right\|}_{2}^{2}$$

Therefore, the update formula of ADMM consists of the minimization of *x*, *y* and the update formula of *z*.

(A3) $$\left\{\begin{array}{l} x^{k+1}=\arg\min_{x}L_{v}\left(x,y^{k},z^{k}\right)\\ y^{k+1}=\arg\min_{y}L_{v}\left(x^{k+1},y,z^{k}\right)\\ z^{k+1}=z^{k}+v\left(Ax^{k+1}+By^{k+1}+b\right) \end{array}\right.$$

In order to ensure the convergence of the formula, there is a saddle point (*x*, *y*, *z*), for any *x*, *y*, *z*, and the following conditions must be satisfied.

(A4) $$L_{v}\left(x^{*},y^{*},z\right)\leq L_{\nu }\left(x^{*},y^{*},z^{*}\right)\leq L_{v}\left(x,y,z^{*}\right)$$

Therefore, Equation (15) is equivalently transformed based on ADMM as follows:

(A5) $$\begin{array}{l} L\left(\left\{v_{k}\right\},\left\{\omega_{k}\right\},\lambda \right)=\alpha \sum_{k}{\left\|\partial_{t}\left[\left(\delta (t)+\frac{j}{\pi t}\right)*v_{k}(t)\right]e^{-j\omega_{k}t}\right\|}_{2}^{2}\\ +\beta \sum_{k}{\left\|\partial_{t}\left[\left(\delta (t)+\frac{j}{\pi t}\right)*v_{k}(t)\right]e^{-j\omega_{k}t}\right\|}_{1}\\ +{\left\|f(t)-\sum_{k}v_{k}(t)+\frac{\lambda (t)}{2}\right\|}_{2}^{2} \end{array}$$

First, we optimize the modal components by fixing and constructing subproblems to minimize the Lagrange function. Using the Fourier isometry, the subproblem expression (Equation (A1)) is obtained, where the L2 norm term is

(A6) $$\alpha \sum_{k}\int_{-\infty }^{\infty }{\left|j\omega \left[1+\mathrm{sgn}\left(\omega +\omega_{k}\right)\right]{\hat{v}}_{k}\left(\omega +\omega_{k}\right)\right|}^{2}d\omega$$

For the positive frequency range, the above formula simplifies as

(A7) $${\left|j\omega \left[1+\mathrm{sgn}\left(\omega +\omega_{k}\right)\right]{\hat{v}}_{k}\left(\omega +\omega_{k}\right)\right|}^{2}={\left|j\omega \cdot 2{\hat{v}}_{k}\left(\omega +\omega_{k}\right)\right|}^{2}=4\omega^{2}{\left|{\hat{v}}_{k}\left(\omega +\omega_{k}\right)\right|}^{2}$$

Similarly, the L1 norm term in Equation (A1) is expressed as

(A8) $$\beta \int_{0}^{\infty }2\left|j\left(\omega -\omega_{k}\right){\hat{v}}_{k}(\omega )\right|d\omega =\beta \int_{0}^{\infty }2\left|\omega -\omega_{k}\right|\left|{\hat{v}}_{k}(\omega )\right|d\omega$$

To solve Equation (A1), we set the objective function as

(A9) $$\begin{array}{l} J\left({\hat{v}}_{k}\right)=\alpha \int_{0}^{\infty }4{\left(\omega -\omega_{k}\right)}^{2}{\left|{\hat{v}}_{k}(\omega )\right|}^{2}d\omega +\beta \int_{0}^{\infty }2\left|\omega -\omega_{k}\right|\left|{\hat{v}}_{k}(\omega )\right|d\omega \\ +\int_{-\infty }^{\infty }{\left|\hat{f}(\omega )-\sum_{i\neq k}{\hat{v}}_{i}(\omega )-{\hat{v}}_{k}(\omega )+\frac{\hat{\lambda }(\omega )}{2}\right|}^{2}d\omega \end{array}$$

Compute the variational derivative with respect to $v_{k}$, as follows:

(A10) $$J=\int_{0}^{\infty }4\alpha {\left(\omega -\omega_{k}\right)}^{2}{\left|{\hat{v}}_{k}\right|}^{2}d\omega +\int_{0}^{\infty }2\beta \left|\omega -\omega_{k}\right|\left|{\hat{v}}_{k}\right|d\omega +\int_{-\infty }^{\infty }{\left|\hat{f}-\sum_{i\neq k}{\hat{v}}_{i}-{\hat{v}}_{k}+\frac{\hat{\lambda }}{2}\right|}^{2}d\omega$$

where the L2 norm term is expressed as

(A11) $$\frac{\delta }{\delta {\hat{v}}_{k}^{*}}\left[4\alpha {\left(\omega -\omega_{k}\right)}^{2}{\left|{\hat{v}}_{k}\right|}^{2}\right]=4\alpha {\left(\omega -\omega_{k}\right)}^{2}{\hat{v}}_{k}$$

The data fidelity term is expressed as

(A12) $$\frac{\delta }{\delta {\hat{v}}_{k}^{*}}{\left|\hat{f}-\sum_{i\neq k}{\hat{v}}_{i}-{\hat{v}}_{k}+\frac{\hat{\lambda }}{2}\right|}^{2}=-\left(\hat{f}-\sum_{i\neq k}{\hat{v}}_{i}-{\hat{v}}_{k}+\frac{\hat{\lambda }}{2}\right)$$

Let $\left|{\hat{v}}_{k}\right|=\sqrt{{\hat{v}}_{k}{\hat{v}}_{k}^{*}}$, the L1 norm term is expressed as

(A13) $$\frac{\delta }{\delta {\hat{v}}_{k}^{*}}\left[2\beta \left|\omega -\omega_{k}\right|\left|{\hat{v}}_{k}\right|\right]=2\beta \left|\omega -\omega_{k}\right|\cdot \frac{{\hat{v}}_{k}}{2\left|{\hat{v}}_{k}\right|}=\beta \left|\omega -\omega_{k}\right|\frac{{\hat{v}}_{k}}{\left|{\hat{v}}_{k}\right|}$$

By combining the above terms, the variational derivative of the objective functional is given by

(A14) $$4\alpha {\left(\omega -\omega_{k}\right)}^{2}{\hat{v}}_{k}+\beta j\left(\omega -\omega_{k}\right){\hat{v}}_{k}-\left(\hat{f}-\sum_{i\neq k}{\hat{v}}_{i}-{\hat{v}}_{k}+\frac{\hat{\lambda }}{2}\right)=0$$

Further simplifying, we get

(A15) $$\left[4\alpha {\left(\omega -\omega_{k}\right)}^{2}+\beta j\left(\omega -\omega_{k}\right)+1\right]{\hat{v}}_{k}=\hat{f}-\sum_{i\neq k}{\hat{v}}_{i}+\frac{\hat{\lambda }}{2}$$

Therefore, we obtain

(A16) $${\hat{v}}_{k}^{n+1}(\omega )=\frac{\hat{f}(\omega )-\sum_{i\neq k}{\hat{v}}_{i}(\omega )+\frac{\hat{\lambda }(\omega )}{2}}{1+4\alpha {\left(\omega -\omega_{k}\right)}^{2}+\beta j\left(\omega -\omega_{k}\right)}$$

Similarly, construct the subproblem for the center frequency, that is,

(A17) $$\omega_{k}^{n+1}=\arg\min_{\omega_{k}}\left\{\alpha \int_{0}^{\infty }{\left(\omega -\omega_{k}\right)}^{2}{\left|{\hat{v}}_{k}(\omega )\right|}^{2}d\omega \right\}$$

By taking the derivative of Equation (A17) and setting it to zero, we obtain the critical points.

(A18) $$\frac{\partial }{\partial \omega_{k}}\left[\alpha \int_{0}^{\infty }{\left(\omega -\omega_{k}\right)}^{2}{\left|{\hat{v}}_{k}(\omega )\right|}^{2}d\omega \right]=-2\alpha \int_{0}^{\infty }\left(\omega -\omega_{k}\right){\left|{\hat{v}}_{k}(\omega )\right|}^{2}d\omega =0$$

Rearranging Equation (A1) yields the following expression:

(A19) $$\int_{0}^{\infty }\left(\omega -\omega_{k}\right){\left|{\hat{v}}_{k}(\omega )\right|}^{2}d\omega =0$$

Upon integrating the aforementioned equation and rearranging terms, we arrive at

(A20) $$\int_{0}^{\infty }\omega {\left|{\hat{v}}_{k}(\omega )\right|}^{2}d\omega =\omega_{k}\int_{0}^{\infty }{\left|{\hat{v}}_{k}(\omega )\right|}^{2}d\omega$$

The final update formula for the central frequency is given by

(A21) $$\omega_{k}^{n+1}=\frac{\int_{0}^{\infty }\omega {\left|{\hat{v}}_{k}(\omega )\right|}^{2}d\omega }{\int_{0}^{\infty }{\left|{\hat{v}}_{k}(\omega )\right|}^{2}d\omega }$$

In summary, through the above derivation, Equations (19) and (20) are obtained.
